# Cervical Cancer in Women With HIV: A Call to Action for Equitable Prevention in Low‐ and Middle‐Income Countries

**DOI:** 10.1155/bmri/1711050

**Published:** 2025-12-07

**Authors:** Jonathan Klutse, Yeena Abla Tay, Dzidzor Aku Attoh, Emmanuel Frimpong Gyekye, Charlotte Borteley Bortey, Esenam Dzifa Buatsi, Charlayne Cherylyn Oppong, Helena Lamptey, Collins Stephen Ahorlu, George Boateng Kyei, Evelyn Yayra Bonney

**Affiliations:** ^1^ Department of Virology, Noguchi Memorial Institute for Medical Research, University of Ghana, Accra, Ghana, ug.edu.gh; ^2^ Department of Immunology, Noguchi Memorial Institute for Medical Research, University of Ghana, Accra, Ghana, ug.edu.gh; ^3^ Department of Epidemiology, Noguchi Memorial Institute for Medical Research, University of Ghana, Accra, Ghana, ug.edu.gh; ^4^ Medical and Scientific Research Centre, University of Ghana Medical Centre, Accra, Ghana, ugmedicalcentre.org; ^5^ Department of Medicine and Molecular Microbiology, Washington University in St. Louis, St. Louis, Missouri, USA, wustl.edu

**Keywords:** cervical cancer, cervical cancer screening, HIV, HPV DNA, human papillomavirus

## Abstract

Cervical cancer is preventable; however, it remains the leading cause of death in low‐ and middle‐income countries (LMICs). Women with HIV (WWHs) have a sixfold higher risk of developing and dying from cervical cancer than women without HIV. Cervical cancer can be prevented by vaccination against high‐risk human papillomaviruses (hrHPVs) and by screening for and treating precancer cervical lesions. While these preventive measures are routinely available to WWHs in developed countries, they are lacking in most LMICs, where the burden of HIV and cervical cancer is the highest. To prevent cervical cancer deaths among WWHs in LMICs, it is imperative to determine the dual burden of HIV and cervical cancer in LMICs. This narrative review synthesized scientific papers and policy documents on the intersection of HIV and cervical cancer in LMICs published between August 2006 and July 2025. We searched PubMed, Scopus, Web of Science, and Google Scholar for articles and official reports from the World Health Organization (WHO) and the US Centers for Disease Control and Prevention (CDC) on cervical cancer burden, prevention strategies, barriers, and outcomes among WWHs. Despite its proven effectiveness, HPV vaccination coverage in LMICs is under 30%, and screening uptake is below 20%. Weak health systems, workforce shortages, stigma, reliance on donor funding, and late‐stage case presentation are major challenges in curbing cervical cancer in LMICs. Urgent political commitment is required to integrate precancer screening and HPV testing into routine HIV care and scale‐up HPV vaccination to achieve the WHO′s triple‐intervention targets to eliminate cervical cancer among WWHs in LMICs.

## 1. Introduction

Cervical cancer (CC) continues to be a global threat to women′s health. It is the fourth most common cancer in women after breast, colorectal, and lung cancers [1] and the most common cancer among women with HIV (WWHs). In 2022, approximately 660,000 women were diagnosed with CC, and over 350,000 died from the disease. Of these deaths, 47% occurred in low‐ and middle‐income countries (LMICs) and 40% in upper middle‐income countries [2], which bear the greatest burden of the disease.

In Ghana, CC is the second most frequent cancer among women in the general population and the second most common cancer among women aged 15–44 years. The World Health Organization (WHO) estimates the incidence of CC in Ghana to be 27/100,000, four times that of the United States, with a mortality rate of 16.9/100,000, nearly eight times that of the United States [3].

Globally, HIV significantly contributes to the CC burden. In 2018, 5.8% of newly diagnosed CC cases globally were in WWHs, and 4.9% of cases were attributable to HIV infection [4]. The regions most affected were Southern and Eastern Africa, where 63.8% and 27.4% of CC cases, respectively, occurred in WWHs [4]. Currently, 85% of women with CC and HIV live in sub‐Saharan Africa (SSA) [4], highlighting HIV′s significant contribution to the CC burden in the region. There is a clear overlap between regions with high HIV prevalence and high CC incidence, underscoring the need for HPV vaccination and cervical cancer screening (CCS) for WWHs. However, these regions have limited access to the available interventions for many women.

CC is primarily caused by infection with oncogenic genotypes known as high‐risk human papillomavirus (hrHPV) [5]. Among these, hrHPV Subtypes 16 and 18 are responsible for approximately 70% of all CC cases. These subtypes are strongly associated with CC, lesions, and preneoplastic dysplasia and contribute to the progression of precancerous lesions to invasive CC [6]. In contrast, low‐risk HPV (LR‐HPV) types are chiefly associated with cutaneous and anogenital warts [7].

Evidence shows that HPV prevalence is consistently higher in WWHs than in HIV‐seronegative women [2, 4, 8]. Furthermore, HPV coinfection with HIV is the most common coinfection in Africa and other countries where both infections are endemic [9].

This narrative review is aimed at providing a comprehensive synthesis of evidence on the dual burden of HIV infection and CC among women in LMICs. It examines the epidemiological burden, underlying biological mechanisms, and systemic barriers that impede prevention, screening, and treatment efforts. By integrating insights from scientific literature and policy reports, this review seeks to guide the development of equitable, evidence‐based strategies, particularly the integration of HPV vaccination and CCS into HIV care, to reduce preventable mortality and advance progress toward the WHO′s elimination targets.

## 2. HIV‐Predisposing Factors for CC

Several studies have implicated immune dysfunction due to HIV infection as the major predisposing factor for HPV infections and CC in WWHs [4, 10, 11]. The greater prevalence of HPV infections in WWHs is linked to immune dysfunction induced by HIV infection, which impairs the rate of HPV clearance and increases its oncogenic risk [12]. Thus, immunocompromised WWHs are less likely to clear hrHPV that causes CC than their seronegative counterparts [13]. Furthermore, the accompanying immune suppression and immune activation with HIV infection increase the risk of developing CC by about sixfold among WWHs [4, 8, 14]. This increased risk persists even in women on antiretroviral therapy (ART) with suppressed viral loads [11]. This underscores the need for enhanced CC prevention strategies, such as prioritizing prophylactic HPV vaccination among WWHs, especially girls with HIV, and initiating ART early to achieve viral suppression, restore immune function, and reduce the risks associated with HPV acquisition and progression. In addition, studies have shown that HIV infection plays an indirect role in oncogenesis, mainly via immune suppression, enhancing the effects of hrHPV, as evident by the association of CC with lower CD4 cell count and no ART among WWHs [13]. In contrast, WWHs with higher CD4+ T‐cells have a lower risk of cervical precancer lesions and cancer [15–17]. Infection with HIV is also associated with more extensive lesions of the cervix, occurring three to five times more frequently in WWHs than in those with no history of HIV [18]. HIV not only increases the risk of CC but also increases the rates of recurrence following treatment for precancerous lesions [19] and decreases the life expectancy [20]. A retrospective study by Chambuso et al. [21] in Tanzania reported a 71.8% prevalence of cervical precancerous lesions in WWHs and 27.3% in HIV‐seronegative women, a 40.5% prevalence of extensive precancerous lesions in WWHs compared to 13.5% in their seronegative counterparts, and an 11% prevalence of invasive CC in WWHs compared to 8% in HIV‐seronegative women. In addition, Stelzle et al. [4] showed that approximately 5% of CCs are attributable to HIV infection and over 40% of women diagnosed with CC from nine southern countries have HIV. CC develops at least two times faster in women with untreated HIV infection than in other women [22], confirming that HIV infection predisposes women to CC.

## 3. Burden of CC Among WWHs in LMICs

The global burden of CC is associated with the heightened risk among WWHs. In contrast to LMICs, CC incidence rates in WWHs in high‐income countries (HICs) have declined over the past three decades due to access to ART and the broader availability of CCS services. Currently, the most affected regions are Southern and Eastern Africa, contributing to 70% of the dual burden of HIV and CC [4]. These regions carry a more significant disease burden due to the substantial HIV‐attributable CC burden, which has added to the existing CC burden. In the African region alone, 85% of CC cases were attributed to HIV as of 2018 [4]. Globally, Eswatini had the highest proportion of women with CC living with HIV. In Stelzle et al.′s [4] meta‐analysis, 11 of the 16 West African countries were part of the 48 countries that contributed to the 5% of CCs attributable to HIV in 2018. In Cameroon, an estimated 22.66% (95% CI: 17.21–29.1) of new CC patients are living with HIV, representing the highest burden in the region. In contrast, Ghana ranks sixth among the 11 assessed West African countries, with an estimated 11.92% (95% CI: 8.56–16.22) of new CC patients living with HIV [4].

## 4. Prevention of CC

Although CC is one of the most easily preventable and curable cancers, it continues to affect many women globally and claims many lives. Its successful prevention is based on early diagnosis and detection of precancerous lesions [23]. Apart from standardized screening, other key recommended preventive initiatives among the general population include HPV vaccination and education about contributing factors to encourage risk avoidance [24]. To reduce the burden of CC, the WHO has tasked all countries to implement the triple‐intervention strategy to eliminate CC as a public health threat by 2030:

• Ninety percent of all girls are vaccinated for HPV before Age 15.
• Seventy percent of all adult women are screened by Age 35 and again by Age 45.
• Ninety percent of women are identified with precancerous lesions treated [25].

The WHO′s strategies include recommendations for both women in the general population and WWHs. CC prevention can be grouped into two major categories:

• Primary prevention: This is intended to prevent cervical neoplasms by preventing HPV infection using vaccination.
• Secondary prevention: The use of standardized screening aiming to detect precancerous cervical lesions and treat these lesions to prevent the development of cancer.

### 4.1. Primary Prevention by Prophylactic HPV Vaccination

The primary CC prevention strategy involves routine prophylactic HPV vaccination among the general population. Currently, four prophylactic HPV vaccines are available: Cervarix, Gardasil, Gardasil 9, and Cecolin. Cervarix and Cecolin are bivalent HPV vaccines, Gardasil is a quadrivalent vaccine, and Gardasil 9 is a nonvalent vaccine. Gardasil (Merck & Co., Kenilworth, New Jersey) [26] and Cervarix (GlaxoSmithKline, Rixensart, Belgium) were the first US FDA‐approved HPV vaccines for hrHPV [27]. These vaccines target HPV‐6 and HPV‐11, two non–high‐risk HPV strains responsible for 90% of anogenital warts, and HPV‐16 and HPV‐18, two hrHPV strains associated with approximately 70% of CC cases [28]. The first batch of HPV vaccines was followed by Gardasil 9 (Merck & Company), which notably targets HPV‐31, HPV‐33, HPV‐45, HPV‐52, and HPV‐58 in addition to HPV‐6, HPV‐11, HPV‐16, and HPV‐18 and is predicted to prevent approximately 90% of CC [29]. The CDC recommends routine HPV vaccination for girls and boys aged 11–12 years [30], while according to the WHO [31], vaccination programs should target girls aged 9–13 years. Vaccinating 90% of eligible girls against HPV is one of the WHO′s triple‐pillar intervention strategies for eliminating CC before 2030. Meeting this and the two other targets by the end of this decade could eliminate CC by 2100, saving over 62 million lives and reducing death by 99% [32].

Literature has shown consistent evidence that in HPV‐naïve women, the bivalent and quadrivalent HPV vaccines containing HPV‐16 and HPV‐18 antigens protect with high efficacy against infection and precancerous cervical lesions associated with these types. The WHO suggests that vaccinating girls aged 9–14 against HPV can prevent up to one‐third of HPV‐related malignancies in Africa [33, 34]. However, these vaccines are not widely available in most parts of Africa [35–37]. Currently, Rwanda, Tanzania, South Africa, and Senegal are among the 15 countries that have included the HPV vaccine in their national immunization programs after successful pilot studies [38]. Nigeria and a few other countries have ongoing pilot immunization programs [37, 39]. In addition, there is no optimal HPV vaccination strategy for long‐term protection against CC in WWHs, particularly those in LMICs, including Ghana. Furthermore, no HPV vaccine studies have been conducted on efficacy specific to people with HIV. Nonetheless, there is evidence of tolerance, safety, and strong immune responses following HPV vaccination in individuals with HIV. One study reported a seroconversion rate of 0.85 in WWHs and 0.91 in HIV‐negative persons aged 13–27 years. A similar seroconversion rate was observed in the study of Gardasil in adult men living with HIV [40]. However, primary prevention through HPV vaccination is less likely to reduce HPV‐related cancers in WWHs because these vaccines only work to prevent initial HPV infection, and most HIV‐infected individuals were not vaccinated against HPV in time to prevent initial anogenital HPV infection.

### 4.2. Secondary Prevention: CCS

Currently, there are three options for CCS: cytology, HPV testing, and cytology–HPV cotesting. Cytology‐based screening remains the most common approach, involving cervical cytological examination using conventional smear or liquid preparation systems [41]. As part of the new guidelines for CCS and treatment, the WHO recommends HPV DNA testing as the primary CCS test for both the general population of women and WWHs. Interestingly, some studies have demonstrated that HPV DNA testing is much more effective than Papanicolaou (Pap) testing and visual inspection with acetic acid (VIA) [42, 43]. A cluster randomized trial involving approximately 100,000 Indian women aged 30–59 years showed that a single round of HPV DNA testing reduced CC‐related mortality by 50% in 8 years. In contrast, Pap testing and VIA did not reduce cancer‐related mortality compared to community control [44].

However, in areas where HPV DNA testing is not available, VIA, visual inspection using Lugol′s iodine (VILI), and Pap tests may continue to be used in accordance with local guidelines. Pap testing, as endorsed in earlier recommendations by the WHO, usually involves a pelvic examination, during which a smear of cervical cells is taken for the Pap smear test. In contrast, using VIA, trained personnel insert an unlubricated bivalve speculum into the vagina to examine the cervix. A halogen focus lamp is used to identify the squamocolumnar junction (SCJ). After clearing any excess mucus with a cotton swab, a 5% acetic acid solution is applied to the cervix for visual inspection. Results are visible 1 min after application. A positive VIA result is characterized by a distinct, dense acetowhite area with regular or irregular margins located near or touching the SCJ in the transformation zone or when the entire cervix or cervical growth turned acetowhite. VILI is conducted similarly, and a positive VILI test is identified by a dense, thick, bright, mustard‐yellow or saffron‐yellow area where iodine is not taken up, observed in the transformation zone, close to or abutting the SCJ, or when the entire cervix or a cervical growth turned densely yellow [18].

For routine cervical screening, HPV testing is recommended every 5 years, cotesting (HPV testing and Pap test) every 5 years, or Pap tests every 3 years should begin at the age of 25 and end at 65 years for those with no abnormal results or severe diagnoses [45]. For WWHs, the guidelines state that screening should start at 21 or several years before Age 25, when the risk of invasive CC among WWHs is more significant than that in the general population. Screening should continue after the age of 65 years, as WWHs have a higher risk of developing CC. For women below Age 30, a Pap test should be done yearly for 3 years and then every 3 years if previous results were normal. For women above 30 years of age, Pap testing alone or cotesting should be performed every 3 years if the initial result is negative.

## 5. Current CCS Recommendations for WWHs

**Figure 1:**
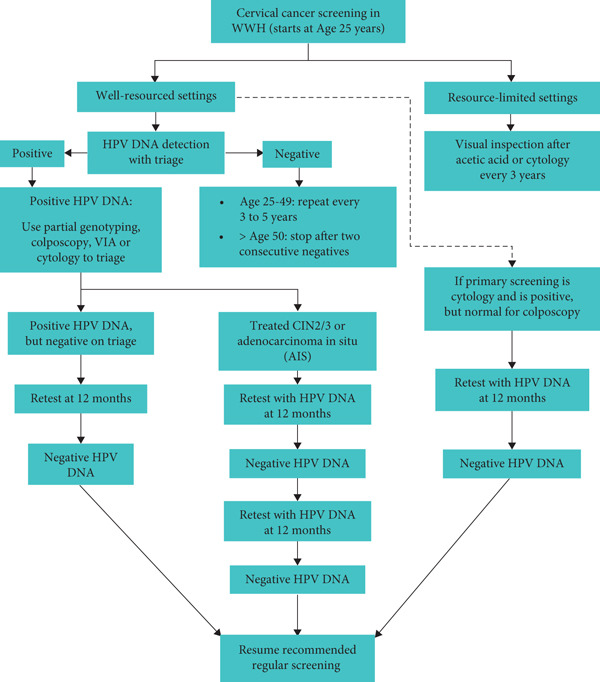
Current WHO cervical cancer screening recommendation for women with HIV.

The WHO′s new guideline on cervical screening among WWHs recommends the use of HPV DNA testing in a screen, triage, and treat approach as the primary CCS test [25]. However, the VIA and Pap tests may continue to be used according to local guidelines in various settings when HPV DNA testing is not operational [25]. A study by Hall et al. [46] among WWHs in Tanzania supports the WHO′s screen, triage, and treat approach. Triaging HIV‐HPV‐positive women before treatment resulted in minimal loss of effectiveness and had a more favorable number needed to treats (NNTs) (19.7–33.0) [46]. In addition, screening using VIA or cytology was less effective than primary HPV, as VIA generated a far higher NNT of 107.5 [46]. In the guidelines, HPV testing–based screening is recommended for women aged 25–49 years every 3–5 years. According to the WHO, HPV DNA testing should be performed regularly among WWHs beginning at the age of 25 years. In this regard, either a healthcare provider or the individual herself can collect the necessary samples [25]. Figure 1 summarizes the current WHO′s recommendation for WWHs.

## 6. Management of CC in WWHs

There are several determinants of the choice of CC therapies, including cancer stage, whether the cancer has metastasized to other body parts, tumor size, and the patient′s age and overall health [24]. Due to the lack of awareness and low uptake of CCS in WWHs, especially in LMICs [47], most women present to clinics with locally advanced CC. Radiotherapy is the primary treatment for these women [28]. This treatment choice is also independent of HIV status. Unfortunately, the lack of radiation equipment, trained professionals, cost differences, and delays due to limited services in public sector hospitals in LMICs have resulted in the choice of surgery over radiation treatment [28]. Shah et al. [48] in their meta‐analysis of retrospective or prospective cohort studies on the chemoradiation treatment of locally advanced CC in WWHs found that

• Eight (8) of 13 studies identified reported no significant differences in treatment outcome by HIV status.
• Six out of eight studies that assessed survival reported no significant difference in survival based on HIV status.
• All four studies assessing treatment response found no significant differences based on HIV status.
• All six studies identified reported no significant differences in toxicity between WWHs and HIV‐negative women.

## 7. Challenges of CC Prevention and Control Among WWHs

CC is preventable in women through HPV vaccination, HPV testing, screening, and treatment of cervical precancers. However, it remains a major public health concern in LMICs, with WWHs being disproportionately affected. The WHO recommends HPV vaccination of girls by Age 15 years, testing, and screening of women starting at age 35 years for women without HIV and age 25 years for WWHs as effective strategies to mitigate this burden. However, in LMICs, where the incidence of CC is significantly higher, many countries, including Ghana, do not have routine national screening and control programs for WWHs. The higher risk of CC among WWHs [49] warrants more targeted screening for HPV infection and precancer screening in this vulnerable population [50]. The lack of structured, population‐based interventions reflects both limited government commitment and weak health systems.

The WHO′s recommendations for cervical precancer screening in WWHs are critical for curbing CC deaths. However, the uptake rate in LMICs remains woefully inadequate due to resource and infrastructure constraints. In HICs, routine screening programs and widespread HPV vaccination have significantly reduced CC incidence and mortality. These settings achieve high vaccine coverage through school‐based delivery systems and ensure access to regular Pap smears and HPV DNA testing within well‐resourced healthcare infrastructures [1]. In LMICs, including SSA, cervical precancer screening coverage ranges from 0.4% to 20.2%, whereas developed countries report coverage exceeding 60% [51, 52]. These stark disparities in cervical precancer screening can be attributed to disparities in countries′ healthcare policies, such as the structure of institutions, access to healthcare, and availability of screening programs across nations [53]. Only 8 of the 49 countries in SSA have successfully implemented national HPV immunization programs for women in the general population [53]. None of these eight countries has implemented national CC prevention programs specifically for WWHs, although WWHs are at a disproportionately higher risk of persistent HPV infection and faster progression to CC. Although some SSA countries have HPV vaccination programs for the general population, WWHs, who should be prioritized, remain underserved, reflecting structural inequities in health policy and program implementation. Additionally, HIV programs in SSA are relatively well established; therefore, the lack of cervical precancer screening and HPV testing as part of routine care for WWHs in these settings represents a missed opportunity for the cost‐effective prevention of CC deaths.

The obstacles to CC prevention and control among WWHs in LMICs remain multifaceted. Limited knowledge of CC and screening services is reinforced by misconceptions and fears surrounding pain, embarrassment, and stigma [54, 55]. In Ghana, the lack of CCS uptake has been attributed to ignorance about the disease, cost of screening, fear of pain, embarrassment, nonavailability of facilities for screening, and the fear of a cancer diagnosis [54, 55]. These challenges further restrict access to CC services for WWHs. Similar patterns have been reported across SSA. In Ethiopia, despite CC being the most common cancer among women, only 34.2% of WWHs are aware of the disease, illustrating the pervasive knowledge gaps that impede early detection [56]. As a result, late‐stage presentations are common, associated with poor prognosis and limited therapeutic options. Deficiencies in infrastructure, shortages of trained personnel, and inadequate governmental investment further undermine prevention and control efforts [57]. For instance, in Malawi, Denny et al. [58] reported that there was only one pathologist, one colposcope, and no facilities for CCS and treatment. The absence or shortage of healthcare workers means that high‐risk populations, such as WWHs, are often not identified until advanced disease, increasing mortality in WWHs. Ndejjo et al. [59] showed that access to health facilities in rural areas of Uganda, where CCS is performed, is often difficult due to a lack of transportation, the cost of transportation, and the long distances to health centers. WWHs often face stigma when accessing healthcare. Mensah et al. [60] showed that most women feared being isolated due to the stigma of HIV. Most often, stigma related to CC screening practices is linked to misconceptions where WWHs feared that screening services might reveal their HIV status. Cultural taboos surrounding gynecological examinations and religious beliefs further complicate access. For example, a survey conducted in Nigeria showed that 43 out of 307 WWHs assessed refused to undergo CCS due to religious denial [61]. Structural barriers such as distance, cost, and lack of skilled workers exacerbate their exclusion from prevention services. These inequities reduce the effectiveness of integrated HIV‐CC prevention strategies, such as “screen‐and‐treat” models within HIV clinics.

Regional comparisons highlight the extent of these disparities. In Latin America, countries such as Brazil and Mexico have expanded HPV vaccination and established national screening programs, although rural–urban inequities persist [62]. In Southeast Asia, Thailand has adopted VIA as a cost‐effective strategy, extending coverage despite resource limitations [63]. These examples demonstrate that innovative, context‐appropriate approaches combined with political commitment can yield measurable gains in CC prevention, especially among WWHs.

However, SSA countries, including Ghana, continue to struggle with systemic weaknesses that hinder similar progress. Inadequate infrastructure, limited human resources, and competing health priorities, such as HIV, tuberculosis, and malaria, divert attention and resources away from CC control. Without targeted interventions, WWHs remain at an elevated risk of preventable morbidity and mortality. Scaling up community awareness, embedding screening within HIV care, improving affordability, and building a stronger health system capacity are essential. Comparative experiences from some LMICs indicate that sustained political commitment and the integration of prevention into existing health programs provide feasible pathways to reduce the CC burden and advance equity in outcomes. For instance, a task‐shifting program implemented in Thailand and Ghana to train midwives and auxiliary nurses in administering VIA demonstrated that trained nurses could maintain high‐performance standards. However, Thailand′s successful transition to a national project was due to strong government commitment and support from healthcare workers, while Ghana′s lack of government support, infrastructure, and trained personnel prevented the scale‐up to a national program despite its feasibility [64]. Generally, many CC prevention programs in LMICs rely on donor funding, such as The President′s Emergency Plan for AIDS Relief (PEPFAR), Merck & Co., and other international donors, which have supported CCS integration into HIV care programs in Zambia and Rwanda [65, 66]. Although these donors play important roles in screening programs, vulnerabilities in program continuity are created if funding is discontinued.

## 8. The Way Forward

The public health threat of CC can be mitigated through CCS and vaccination, as recommended by the WHO. Despite this, WWHs in LMICs, including Ghana, continue to bear a disproportionate disease burden due to the unavailability of national CCS and vaccination programs for women in the general population, let alone for WWHs. The unavailability of structured population‐based policies for CC prevention may reflect a lack of governmental support and poor healthcare systems. Other factors may include inadequate funding, low awareness and education on the disease, lack of human resources, including oncologists and gynecologists, and the high cost of screening services.

To address these challenges, it is critical to integrate CCS into routine HIV care. HIV clinicians, researchers, and ministries of health in LMICs need to collaborate to undertake implementation studies to assess the barriers and opportunities to integrate CCS into routine HIV care. Studies are needed to determine the acceptability and reliability of VIA and Pap smears for screening in areas where HPV DNA testing cannot be immediately implemented. Due to the lack of oncologists and gynecologists in most LMICs, studies assessing the feasibility of self‐sampling for HPV DNA testing over healthcare worker sampling are required.

Currently, the Implementing Cervical Cancer Screening Among Women Living with HIV in Ghana (I‐CERV‐GH) project, funded by Expertise France through L′Initiative, is undertaking a pilot study to determine how CCS can be integrated into routine care for WWHs in Ghana, potentially allowing same‐day testing and treatment. This work is being carried out in collaboration with the Cervical Cancer Prevention and Training Centre (CCPTC) at the Catholic Hospital, Battor. The CCPTC is a nonprofit center and the only health facility in Ghana that screens, treats, and trains healthcare professionals across Ghana and neighboring countries. In view of this, other funding agencies are invited to fund the work at CCPTC, in terms of sponsoring trainees, extending community outreaches of the center to other parts of the country, and scaling up studies such as the one undertaken by I‐CERV‐GH in Ghana and other LMICs.

## 9. Conclusion

CC remains a significant cause of death globally, especially among WWHs in LMICs, including Ghana, where most of the disease burden is concentrated. This has been chiefly attributed to ignorance of the disease among WWHs, leading to a low uptake of CC services. In addition, the unavailability of systematic national immunization and screening programs in LMICs, which carry a significant disease burden, has exacerbated the disease incidence. Therefore, it is essential to establish comprehensive national CCS programs for women, especially WWHs, across SSA.

## Disclosure

The content is solely the responsibility of the authors and does not represent the official views of the funders.

## Conflicts of Interest

The authors declare no conflicts of interest.

## Author Contributions

All the authors listed, J.K., E.F.G., D.A.A., C.B.B., Y.A.T., E.D.B., and E.Y.B., conceptualized this paper, with J.K., E.F.G., D.A.A., C.B.B., and Y.A.T. writing on a specific theme. J.K. took the lead in collating and organizing the manuscript draft. E.D.B., H.L., C.S.A., and G.B.K. provided intellectual input, reviewed the manuscript, and offered key revisions that improved its overall quality. E.Y.B. made significant corrections, reviewed and supervised the manuscript writing, and contributed to the final version of the manuscript. All individuals mentioned above have contributed directly and intellectually to this work.

## Funding

This article is part of the I‐CERV‐GH project, funded by Expertise France through L′Initiative (Grant No. 23‐SB0707). E.Y.B. was supported by the National Institute of Allergy and Infectious Diseases of the National Institutes of Health under the Award Number R21AI174880. This work was also supported by the EDCTP2 program supported by the European Union (Grant No. TMA2017SF‐1955, H‐CRIS) awarded to G.B.K.

## Data Availability

Data sharing is not applicable to this article because no datasets were generated or analyzed during the current study.
